# A qualitative study exploring factors associated with mothers’ decisions to formula-feed their infants in Newfoundland and Labrador, Canada

**DOI:** 10.1186/1471-2458-13-645

**Published:** 2013-07-12

**Authors:** Kimberly Bonia, Laurie Twells, Beth Halfyard, Valerie Ludlow, Leigh Anne Newhook, Janet Murphy-Goodridge

**Affiliations:** 1 Research and Evaluation, Newfoundland and Labrador Centre for Health Information, 70 O’Leary Avenue, St. John’s, Newfoundland and Labrador A1B 2C7, Canada; 2 School of Pharmacy and Faculty of Medicine, Memorial University Newfoundland, 300 Prince Philip Drive, St. John’s, Newfoundland and Labrador A1B 3V6, Canada; 3 Faculty of Medicine, Memorial University of Newfoundland, St. John’s, Newfoundland and Labrador A1B 3C7, Canada; 4 Janeway Pediatric Research Unit, Discipline of Pediatrics, Memorial University of Newfoundland, St. John’s, Newfoundland and Labrador A1B 3C7, Canada; 5 Newfoundland and Labrador Provincial Perinatal Program, Janeway Children’s Health & Rehabilitation Centre, St. John’s, Newfoundland and Labrador A1B 3V6, Canada

**Keywords:** Formula feeding, Public Health, Newfoundland and Labrador, Canada, Infant feeding decisions

## Abstract

**Background:**

Breastfeeding has numerous health benefits. In 2010, the province of Newfoundland and Labrador had the lowest breastfeeding initiation rate (64.0%) in Canada. Formula feeding is associated with well-known health risks. Exclusive formula feeding is the “cultural norm” in some regions of the province. Women appear resistant to changing their infant feeding behaviors and remain committed to their decision to formula-feed. The primary aim of this qualitative study was to examine individual factors that shaped mothers’ decisions to formula-feed their infants. Nineteen mothers who were currently formula feeding their children participated in the study.

**Methods:**

Qualitative research in the form of focus groups was conducted in three communities in the province in 2010. A thematic content analysis identified the main themes that influenced mothers’ decisions to formula-feed their infants.

**Results:**

The main themes included issues concerning the support needed to breastfeed, the convenience associated with formula feeding, and the embarrassment surrounding breastfeeding in public.

**Conclusions:**

These findings help to better understand why mothers choose formula feeding over breastfeeding and may help to inform the development of public health interventions targeted at this population of mothers.

## Background

Breast milk has numerous well-known health benefits [1,2]. Breastfeeding provides protection from many diseases and reduces health risks for both mother [3-9] and child [2,10]. Compared to formula feeding, breastfeeding protects against childhood conditions such as gastroenteritis, respiratory tract infections, otitis media, atopic dermatitis, asthma and sudden infant death syndrome [2] and has also been shown to improve cognitive development in children [11] and reduce the risk of developing childhood obesity [12-14]. Formula feeding is associated with well-known health risks [3,9,15,16]. The burden of suboptimal breastfeeding has been estimated at 13 billion dollars in the United States [17]. As a result, the World Health Organization (WHO) recommends infants be exclusively breastfed for six months with a goal of breastfeeding for up to two years of age and beyond [1]. Although the benefits of breastfeeding are well-evidenced, there are still wide variations globally in initiation and duration rates [18,19]. With the development of a commercial industry that manufactures and heavily markets cow’s milk formula and due to its perceived benefits of convenience and equivalent nutrition, formula feeding has become the norm in many western, industrialized countries over the past several generations [20]. Increasing access to technology such as Facebook, blogs, mobile applications and YouTube videos has allowed the infant formula industry to more heavily market their products to expectant and new mothers often violating the WHO International Code of Marketing of Breast Milk Substitutes [21].

In the Canadian province of Newfoundland and Labrador (NL), the initiation rate for breastfeeding in 2010 was the lowest in Canada (64.0% compared to the Canadian average of 87.1%) [22]. In addition, exclusive breastfeeding rates to six months were also low at 15.4%, compared to the average Canadian rate of 25.9% [22]. More concerning is that within the province of NL, there are very wide regional variations in initiation rates. The most recent population data demonstrate that in 2012, breastfeeding initiation varied within the province from a low of 44.0% to 74.4% [23]. In addition, despite increased breastfeeding promotion and support initiatives over the last ten years, initiation rates in NL are increasing very slowly [23]. In some regions of the province, the rates of formula feeding are very high, even though many women appear to know the benefits of breastfeeding. Women in these regions appear resistant to changing their choice of infant feeding method and often rationalize their decisions to formula-feed [24,25].

There has been limited research in NL that focuses on gaining a better understanding of why so many mothers choose to formula-feed their infants [26,27]. However, a number of studies suggest socio-demographic factors are associated with the choice to formula-feed. These include younger age, lower education and income, being a single parent and a current smoker [28-33]. Although many of these same women seem to be knowledgeable about the benefits of breastfeeding, young low-income women tend to be greatly influenced by the attitudes held by their social environment [34,35] as well as the attitudes and support, or lack of support, from their own mothers [30]. Many of these factors are difficult to modify and what is needed is a better understanding of why mothers with these characteristics make their decision to formula-feed in order to develop targeted interventions.

In addition, given the well-known risks of formula feeding for mother and baby [3,9,15,16] from a public health perspective, it is critical to better understand the factors that influence these decisions with the overall goal of promoting a behavior that will improve the health of both the baby and mother [2-14,16]. The primary objective of the current study was to examine individual factors that shaped mothers’ decisions to formula-feed their infants.

## Methods

### The setting

The Breastfeeding Research Group (BFRG), under the umbrella of the Baby-Friendly Council of NL (formerly the Breastfeeding Coalition of NL), has established a multidisciplinary team to develop research focused on infant feeding in NL [14,24,25]. The BFRG is an experienced team of researchers, health care providers, research users and policy makers who are interested in understanding why breastfeeding rates are so low in NL, with the long-term goal of improving rates of initiation and duration and positively impacting the population’s overall health and well-being. Part of the BFRG’s program of research is collecting and analyzing quantitative data to identify predictors for intent, initiation, and duration of breastfeeding in NL. Additionally, in order to provide a more complete picture of the infant feeding concerns in NL, the BFRG conducted a qualitative study to understand reasons why mothers make formula feeding choices.

### Sample

During the summer of 2010, a purposive sampling of mothers who were formula feeding their infants was conducted in the eastern region of NL (the most populated area of the province including the capital city, St. John’s). In particular, one urban and two rural sites were targeted. Potential participants were invited by staff from family resource programs to participate in an organized focus group. Family resource programs offer resources and activities related to child development and parental support to low-income families or those with financial and social challenges. A package including the date and time of the focus group was given to potential participants. If interested, the mothers could attend the session. At the end of the session, a $20 gift card to a local supermarket was given to participants as a thank you for their participation. Mothers were eligible for the study if they were over 18-years-of-age, had delivered a full-term healthy infant, and were formula feeding their youngest/only child.

### Methodology

This was a qualitative study using focus groups. Focus groups provide an opportunity for women of similar backgrounds and living situation to speak freely and openly about their experiences [36]. Contrary to the view that interviews provide a better forum for the discussion of sensitive and personal issues, the focus group environment provides the social peer support needed to facilitate the sharing of personal experience [37,38].

Two focus groups and one interview took place between July and August 2010 in two rural regions and one urban area of NL. Ideally, a focus group should consist of 6 to 8 participants in order to generate enough material and not to overwhelm the facilitator [37]. In this study, there were 6 participants in the urban focus group, 12 in the first rural group and 1 in the final rural interview. A semi-structured interview guide was used to direct the conversations. Open discussions and questions were posed in a non-judgmental manner (“Why did you choose to formula-feed your baby?”) rather than one with negative connotations (“Why did you choose not to breastfeed your baby?”). The sessions were conducted by an experienced qualitative researcher, who collected demographic information on a pre-approved form, introduced the study to the participants, requested their permission to record the sessions, assured the participants that their identifying information would be removed and encouraged participants to discuss pertinent topics relating to their formula feeding experience. The sessions lasted between 30 and 50 minutes and were audio recorded and transcribed. Information that might identify participants was not included in subsequent reports.

### Data analysis

A thematic content analysis was used to analyze focus group and interview transcripts through inductive coding described by Strauss and Corbin [39]. QSR International Nvivo 8 qualitative data analysis software [40] assisted in the open coding and analysis of the transcripts. Open coding involves reading through each transcript to determine the overall content. Codes were created based on their similarities. Subcategories were developed from the open coding categories and were linked with other categories and subcategories, depending on context. From the subcategories, themes were developed, while making note of significant statements. The codes and themes developed from the focus groups and interview were compared within each transcript as well as between transcripts. The analysis was conducted by two researchers who independently identified and categorized themes. On sharing their findings, the researchers highlighted common themes that helped explain why mothers chose to formula-feed their infants. The researchers discussed the themes and came to a consensus on what were the overarching themes in the study [41]. Feedback from the research team was also incorporated where appropriate.

### Ethics

Ethical approval was obtained from the Human Investigation Committee of Memorial University of NL in the spring of 2010. An approved consent form was signed by each participant at the start of each session after providing the participants with the opportunity to ask questions.

## Results

### Participant characteristics

There were a total of 19 participants. Thirteen of the women who participated were living in a rural community and six were living in an urban centre of the province. The women ranged in age from 20 to 36 years with an average age of 26 years. The children being formula-fed ranged from one month to just over two-years-of-age. Almost all the women provided their marital status; nine were single mothers and nine were married or living with a common-law partner. The majority (n = 16) of women had some high school education or had graduated from high school. In 2005, only 25% of Canadian women reported similar education levels [42] signifying that the current study population was a particularly vulnerable population. Of those who provided their income (n = 17), the majority had an annual income ranging from $10,000 - $20,000, compared to 24.7% of NL households who reported similar annual incomes [43]. Five of the women were working either part-time or full-time. Thirteen mothers reported being unemployed or using social assistance. The majority (n = 11) of women were smokers, compared to 25% of NL mothers who smoked in 2005 [42]. The women that took part in the current study had all chosen to formula-feed their most recent child. Overall, the sample of mothers participating in the study was socio-economically below the NL/Canadian average in recent years. Participant socio-demographic information is found in Table 1.

**Table 1:** Participant socio-demographics

Socio-demographic (n = 19)^*^		
**Mother’s Age (years)**	Range	20-36
	Mean	26
**Child’s approximate age (months)**	Range	1-24
**Geographic area**	Living in rural area	13
	Living in urban area	6
Marital status (n = 18)^*^	Single	9
	Married or common-law	9
**Level of education**	Some high school	6
	Graduated from high school	10
	Some vocational education	1
	Graduated vocational education	2
Annual income (n = 17)*	Less than $5,000	3
	Between $5,000-$10,000	1
	$10,000-$20,000	7
	$20,000-$30,000	4
	$30,000-$40,000	2
Employment status (n = 18)*	Working part-time	2
	Working full-time	3
	Unemployed/using social assistance	13
**Smoking status**	Smoker	11
	Non-smoker	8

Notes: * There were a total of 19 study participants who answered demographic information. Of those 18 answered marital status, 18 answered employment status and 17 answered the annual income questions.

### Decision to formula-feed

Before encouraging the participants to discuss factors that influenced their decision to formula-feed, women were asked when they made this decision. Most mothers had decided to formula-feed their babies as soon as they found out they were pregnant or early in pregnancy. The majority of mothers indicated they had already made up their mind regarding how they would feed their baby before visiting their physicians. However, most women stated that they had talked to their physicians about infant feeding, and that their physicians were often the initiators of this discussion.

### Knowledge of breastfeeding

The women were also asked what they knew about breastfeeding. Some women agreed that breast is best, better and healthier for their babies compared to any other feeding option and they expressed this view indirectly, "*Well, they say it's [breast milk] better for the baby…and healthier.*" However, most were of the opinion that formula had the same nutrients and was just as good as or better than breast milk. One mother reasoned, “*My kid was better off on formula…she’s getting more. She’s getting exactly what she needs*”. She continued by explaining that she does not eat healthy food and therefore, her baby is getting more nutrition from formula than if she was breastfed. Another pointed out that her formula-fed baby does not get as many ear infections as another child who was breastfed. Such comments provide some evidence that these mothers have been told or are educated to believe that breast is best. However, they rationalize their own behavior of choosing to formula-feed by voicing the opinion that formula is just as good as, or perhaps even better than breast milk. This contradiction may suggest that mothers, due to health promotion efforts, say that breast is best but do not actually believe it. One participant pointed out that formula includes many additional vitamins (not found in breast milk) and supplements such as vitamin D, Omega 3 and Omega 6. She explained, “*You have to buy vitamin D because the baby doesn’t get it [in breast milk]*”. Another participant voiced her opinion by saying, *“…if it’s on the shelf then obviously it meets regulations”.*

Participants were then asked why they chose to formula-feed their children and what factors had the most significant impact on this decision. Three main themes emerged. These women made the decision to formula-feed their infants due to *issues concerning infant feeding support (f*or example, a lack of support from their mothers, and a desire for partners to want to support them and to be involved in the feeding process). In addition, the perceived *convenience* associated with formula feeding, and the *embarrassment* of breastfeeding in public influenced their decision to formula-feed their infants.

### Issues concerning infant feeding support

#### Grandmothers

Most participants indicated that their own mothers played an important role in their decision-making around infant feeding. All participants indicated that their mothers did not breastfeed them as babies. Some women suggested that when they wanted to talk with their mothers about breastfeeding many of their mothers did not want to discuss the idea. As noted by the women, without the support of family, specifically their mothers, it is difficult to follow through. “*…my mom said, Now, we're not even going to talk about that. You're not going at it*”. Another participant suggested, “*I guess maybe if my mom had breastfed the support would have been there*”.

#### Partners

Many of the women suggested that their partners played an important role in influencing their choice of infant feeding method. These participants believed that their partner felt left out of the changes taking place throughout nine months of pregnancy, and suggested that their partners would be unable to feed the baby if they had breastfed. One mother shared what her partner said to her,

*“For nine months you got to experience everything…the first kicks while she was in you…what did I get to do?* [We went] *to your doctor's appointments…and now I'm sitting back and she's born and I can't even hold her when she's hungry. I can't settle her down because the simple fact is she needs you to feed her”.*

Sharing responsibility of the babies’ care was thought to be not only helpful but also an obligation that should be shared between the parents. The convenience associated with the ability to pass the child onto another person for feeding was thought to benefit the mother’s general well-being.

### Convenience

A second theme identified by the mothers was the perceived convenience associated with formula feeding and the inconvenience of breastfeeding. Some women viewed formula feeding as giving them independence so that they could sleep, do other household chores, look after other children or go outside the home and not worry about having to be home at a certain time to feed the baby. *“There are the tins of formula and now I can go and have a bath. I can go down the road for six hours if I want”.*

Mothers voiced concern that it was inconvenient to have to look for a private place to breastfeed or to have to situate yourself for breastfeeding, *“I got the baby out. I got myself all set up and now you’re latching the baby, and then it’s 20 minutes on this boob, and 20 minutes on [that one]. You know what? Make the formula, you put it in their mouth, the bottle is gone in 15 minutes”.*

### Embarrassment of breastfeeding in public

A common theme discussed by nearly all the participants was the embarrassment associated with breastfeeding in public. While participants stated they did not feel comfortable breastfeeding in public, they shared admiration and support for women who do. “*Personally, I would be a little bit shy of it, but I think…that takes a lot of guts and I wouldn’t be able to do it*”.

Most mothers suggested that going to a private room or covering up is more appropriate in public places than breastfeeding openly. One woman suggested that bottle-feeding is more acceptable in public places “*…taking out the bottle and mixing the formula together is much more acceptable than me sitting out somewhere [breastfeeding the baby]*”.

Related to breastfeeding in public was the sexualization associated with breastfeeding, which was discussed by some of the women in each of the focus groups and interview. One mother made the comment, “*Yes, there are dirty people out there”.* One participant acknowledged society’s influence on the sexualization of breastfeeding, “*Society is what made it [breastfeeding] sexual*”, placing blame on the public.

### Urban and rural differences

While the previous themes were common among both urban and rural participants, there were some differences. Although we did not specifically ask how old participants were when they had their first child, participants from rural areas stated that their infant feeding decision may have been based upon the fact that they were young (some as young as 17-years-of-age) when they had their first child.

Rural participants suggested that many of their health care providers did not discuss breastfeeding with them. One participant stated that her physician told her they were going to talk about breastfeeding, but she quickly responded by saying that she did not want to talk about it and he left it at that.

Most of the urban participants suggested that discussion with and information provided by health care providers is overwhelming. Many participants agreed that health care providers in urban areas are “*pushy*” and overly encourage breastfeeding. One participant stated that she chose to formula-feed her baby out of spite because she felt that breastfeeding was being pushed on her.

## Discussion

NL is one of the least populated Canadian provinces [44] and is fairly large in terms of geographic area with many rural communities having strong traditional roots. Breastfeeding initiation and duration rates in NL, although having improved over the last 50 years, have increased very slowly and appear to have plateaued [23].

Our study found that the belief that breastfeeding is a natural and traditional part of motherhood has been replaced with the belief that formula is as good for babies as breast milk. This concept has also been described in an Australian study involving a comparison of mothers who breastfed and those who did not breastfeed their infants [45]. Similar to the participants in our study, the Australian women rationalized their decision to formula-feed their infants by explaining that if formula was not healthy for infants then it would not be approved for consumption. Our participants also suggested that formula may be even more beneficial than breast milk as the producers of formula are adding extra vitamins to assist in the development and growth of infants. Some mothers from our study voiced a common misconception that formula provides better nutrition than breast milk especially when the mother did not eat nutritional foods.

The three most significant themes in this study were the *issues concerning infant feeding support* from grandmothers and partners, the *convenience* of formula feeding over breastfeeding, and the *embarrassment of breastfeeding in public*.

The first theme, *issues concerning infant feeding support,* significantly influenced the mothers’ decision to formula-feed rather than breastfeed. As with other studies [26,30,32,46] on decision-making around infant feeding, the most influential individuals mothers often turn to for support are their own mothers and their partners. As all of the participants’ mothers had formula-fed their own children and did not have the practical knowledge about and experience with breastfeeding, it was suggested that their mothers may have felt unable to provide support to their daughters [30,47-49]. Engaging grandmothers in the discussion of infant feeding practices is important. This is not a new finding, but emphasizes the important role grandmothers may play in a new mother’s choice of infant feeding method. A number of studies published by Grassley suggest that the experience of grandmothers needs to be acknowledged but that their knowledge can be enhanced along with their support for breastfeeding [47-49]. In addition, developing interventions that empower grandmothers to be more involved and active in supporting their daughters to breastfeed will have positive effects.

Many of the mothers in this study indicated that their partners would have felt left out of feeding their infant. One participant stated that her partner had told her, “*You got to experience everything*”. Morrison, et al. also suggests that if the mother breastfeeds then the grandparents and partner feel left out of infant feeding [30]. Avery and Magnus reported that fathers of infants who were formula-fed felt confident that they could care for their children while the mother was unavailable or away [33]. Like our study, they also found that women were concerned about the father’s involvement with feedings and bonding between the father and child [33].

Earle [29] and Arora, McJunkin, Wehrer and Kuhn [46] suggest that many women make infant feeding decisions before or early in pregnancy and this was also true for women in our study. Many women indicated that they had made this decision early in their pregnancy, often before their health care provider had talked to them about infant feeding. This may indicate that earlier health care provider intervention is needed to promote informed decision-making about breastfeeding, potentially before pregnancy but in the early childbearing years.

While support from partners and grandmothers was an important influencing factor described by participants in our study, the convenience of formula feeding and embarrassment associated with breastfeeding in public were also cited as important influences on their decision to formula-feed. A desire to share feeding responsibilities and the independence associated with formula feeding were found in the literature as well [28,30-33]. The belief that breastfeeding is not convenient for a woman’s lifestyle was also described in the literature. In studies from the United States and Australia, women thought that breastfeeding was time consuming and that formula feeding gave them the opportunity to spend time away from the children [32,50]. As alluded to in our study and in Howett’s study, many women perceive that formula feeding may bring increased independence. They are able to go shopping or run errands and leave the baby with another person [51].

The attitude that breastfeeding in public is not only embarrassing but unacceptable was a common belief held by many of our participants and is reported in the literature [26,28,30,32,33,51]. In Howett’s study, participants felt that breastfeeding in public was the equivalent to ‘*indecent exposure*’ (p. 103) [51]. Most participants in our study felt it was necessary for women to cover up or go to a private area to breastfeed when out in public, but suggested that they would be comfortable breastfeeding in the privacy of their own homes.

The most significant implication from this research study is that the three main themes found to influence the mothers’ decision to formula-feed their babies are potentially modifiable and lend themselves to targeted interventions aimed at this population. As a result, we suggest several policy implications, outlined according to the three main themes that emerged from our findings, some of which are currently being undertaken by the BFRG as a result of this research.

### Issues concerning support

#### Mothers and grandmothers

Although grandmothers in this study could not provide breastfeeding support based on their own experiences, educating grandmothers on the benefits of breastfeeding may be an important first step. Teaching grandmothers practical advice and other ways to help support breastfeeding daughters may improve breastfeeding rates in this population. Further research in this area is currently underway through the BFRG. A qualitative study to explore the experiences of grandmother’s infant feeding decisions is being conducted. Preliminary results support the implications for one possible intervention in the form of including grandmothers in prenatal infant feeding education.

#### Partners

Interventions in the form of education that provides partners and mothers with the knowledge of how partners may support a breastfeeding mother but continue to build a nurturing relationship with their infant may be an important addition to prenatal education. For example, partners can provide support by encouraging and assisting in breastfeeding and in meeting the baby’s myriad of physical and emotional needs beyond feeding. Partners may also provide support in the form of other activities including housework and child care. Mothers may also express breast milk once breastfeeding is well established and partners may choose to participate in the feeding experience.

#### Health care providers

In regions where formula feeding is predominant, health care providers may assist in initiating the conversation about infant feeding choice early in pregnancy in a non-threatening, non-judgmental manner. Pre-natal visits provide opportunities to share information to facilitate an informed decision, explore the mother’s concerns and beliefs and to link with community-based support programs. Health care providers may need training and tools to provide more effective education and support to their clients. A physician breastfeeding tool kit is underway in NL that recommends asking, “*What do you know about breastfeeding?*” rather than the more direct question of “*Are you going to breastfeed?*” which can often cause mothers to be defensive [52].

### Convenience

Convenience associated with formula feeding is a belief that was held by the women in this study. Convenience is inherently linked to embarrassment of breastfeeding in public. In this study women suggest that it is more convenient to formula-feed their infants because it is not necessary to go to a private room or cover up when feeding in public. This finding lends itself to interventions focused on ensuring mothers feel safe and comfortable breastfeeding in public places. Improved public awareness and social acceptability of breastfeeding in public should be encouraged at a policy level. This could include public awareness campaigns including television and radio advertisements, social media and posters that promote mothers feeding in public places as normal and culturally acceptable.

### Embarrassment of breastfeeding in public

Similar to the belief held by mothers about convenience, embarrassment of breastfeeding in public may be addressed through public awareness campaigns. This intervention may target attitudes held by much of society. Again, this may include public awareness campaigns in the form of television and radio commercials, and posters. Also important is the accessibility to family-friendly public spaces, including comfortable and clean areas devoted to breastfeeding and other related child care needs.

Mother-to-mother support may also help to build confidence and self-esteem in breastfeeding mothers. In particular, this can be useful for women who do not have a breastfeeding role model or the support necessary for successful breastfeeding. In addition, there is a need for a change in attitude towards breastfeeding from an early age, for example providing breastfeeding education in the school environment. If young children learn that breastfeeding is a natural way for mammals to feed their young, the notion may be expanded to include humans, thereby normalizing breastfeeding in everyday life. The perceived convenience associated with formula feeding and the embarrassment associated with breastfeeding in public highlights the potential for a public health focus. Many of the interventions recommended above are aimed at improving the social acceptability of breastfeeding in public.

### Study limitations

A limitation of this study was that it was conducted on a small number of participants. One group became an interview since only one mother participated in a pre-arranged focus group; however, the information (as a rural participant) was mirrored by the stories of the other rural group in which twelve people took part. The findings cannot be statistically generalized to larger populations; however, the results in this study were supported by similar larger studies conducted elsewhere. Another possible limitation is that due to the nature of voluntary participation, we may have spoken to women who were confident in their decision to formula-feed and we may have missed factors important to women who were less confident or ambivalent about infant feeding methods. A third limitation is that this research limited its focus to mothers. We focused on mothers because in our society, it is women who usually make infant feeding decisions. We recognize the importance of hearing from both parents and from others who provide support to mothers. We recommend that further research be conducted with diverse families, for example, single fathers, homosexual couples, or other caregivers.

This research question focuses attention on personal attitudes and beliefs about infant feeding; however, as the research team analyzed the data it was clear that the choice of infant feeding method is influenced by a complex web of factors that include the social determinants of health such as marital status, level of education and income, access to prenatal care, and social supports that include family, partner, the health system as well as cultural influences and societal acceptability of breastfeeding in public.

## Conclusions

Our study provides a better understanding of why a formula feeding culture exists in some areas of NL in particular in mothers with low levels of education and income. The issues concerning the support of grandmothers and the desire for support from partners to assist in infant feeding influenced the mothers’ decision to formula-feed. Feelings that formula feeding was more convenient and acceptable in public influenced the mothers’ decision. These findings will help inform public health initiatives aimed at prenatal education and support, health professional education, increasing public awareness and acceptability of breastfeeding. It is recommended that breastfeeding education be directed at mothers, partners, families, health care providers and communities.

## Competing interests

The authors declare that they have no competing interests.

## Authors' contributions

LT and LN were responsible for the initial study concept and design. KB carried out the data collection and drafted the initial manuscript with input from all authors. KB and VL conducted the thematic analysis. BH provided editing of the initial manuscript. KB and LT worked on critical revisions for resubmission. All authors read and approved the final manuscript.

## Pre-publication history

The pre-publication history for this paper can be accessed here:

http://www.biomedcentral.com/1471-2458/13/645/prepub
